# Secondary Autoimmune Dermatological Disorders Induced by Multiple Sclerosis Biological Immunotherapy Agents: A Systematic Review of Case Reports

**DOI:** 10.5812/ijpr-166426

**Published:** 2025-12-02

**Authors:** Mohammad Ali Sahraian, Shahboddin Emami, Sara Ataei, Fahime Nasr Esfahani, Nasibeh Ghalandari

**Affiliations:** 1 Department of Neurology, Multiple Sclerosis Research Center, Neuroscience Institute, Sina Hospital, School of Medicine, Tehran University of Medical Sciences, Tehran, Iran; 2 Department of Clinical Pharmacy, School of Pharmacy, Hamadan University of Medical Sciences, Hamadan, Iran; 3 Tehran University of Medical Sciences, Tehran, Iran

**Keywords:** Multiple Sclerosis, Secondary Autoimmune Disease, Dermatologic Disorders, Biological Medication

## Abstract

**Context:**

Multiple sclerosis (MS) is a devastating autoimmune neurodegenerative disease, for which disease-modifying drugs (DMDs) have been associated with secondary autoimmune dermatological disorders.

**Objectives:**

This systematic review of case reports seeks to examine documented case reports involving biological medications utilized in managing MS attacks and disease progression that correlate with such dermatological complications.

**Evidence Acquisition:**

A systematic search was conducted in the Google Scholar, Scopus, and PubMed databases for studies published until January 2024. The search strategy employed combinations of keywords such as “multiple sclerosis” with specific biological agents (“Natalizumab” OR “Ocrelizumab” OR “Rituximab” OR “Alemtuzumab” OR “Ofatumumab” OR “Ublituximab”) and “case report”, incorporating relevant Medical Subject Headings (MeSH) terms. All articles, if full texts were available, on case reports and case series of autoimmune dermatological complications of biological medication of MS were analyzed. The quality of the case reports was evaluated using the Joanna Briggs Institute (JBI) critical appraisal checklist.

**Results:**

A total of 19 articles fulfilled the inclusion criteria and were included in this review. The highest frequency of secondary autoimmune complications was documented with alemtuzumab administration, whereas rituximab demonstrated the lowest incidence of dermal autoimmune manifestations in MS patients.

**Conclusions:**

The employed injectable MS immunotherapies demonstrate various autoimmune adverse reactions that have been documented across numerous case reports. This review examines different categories of secondary autoimmune complications and explores the theoretical mechanisms underlying their development.

## 1. Context

Multiple sclerosis (MS) is a chronic autoimmune disorder of the central nervous system (CNS), defined by immune-mediated demyelination and progressive neurodegeneration (1). The exact etiology of MS remains incompletely characterized, though interactions between genetic predisposition, environmental factors, and immune system dysfunction are considered contributory (2, 3). The immune system attacks myelin, leading to inflammation and damage, which in turn causes neurological symptoms and nerve apoptosis (4). The implementation of disease-modifying therapies (DMTs) in MS treatment has been linked to the emergence of secondary autoimmune diseases (SADs), representing an unexpected therapeutic complication (5). However, as the utilization of these immunotherapies expands, there is an increasing awareness of potential adverse effects (6). Contemporary research has offered post-marketing insights, examining novel adverse effects of oral MS therapies, highlighting the importance of comprehensive understanding regarding treatment consequences (7, 8). This demonstrates the disease’s intricacy and the need for continued research to better understand its etiology and develop effective treatment plans. The development of secondary autoimmune dermatological conditions in patients receiving MS immunotherapy represents an additional complication of concern (9). Immunotherapy medications employed in MS treatment, including alemtuzumab, may result in secondary autoimmune manifestations (10). However, there is potential for the use of skin-induced immune tolerance, particularly through the use of dermal dendritic cells, in the treatment of MS (11). Recent studies further emphasize immune response modifications in MS patients, which may contextualize these dermatological risks (12). Additionally, associated conditions like spasticity treatments (13) and sleep disorders (14) highlight the multifaceted nature of MS complications.

## 2. Objectives

The present study aims to analyze existing literature to provide a comprehensive review of the current knowledge on the underlying mechanisms and risk factors associated with this complex interplay.

## 3. Evidence Acquisition

### 3.1. Data Sources

The present review focuses on identifying and analyzing autoimmune dermatological disorders associated with DMTs used to manage MS. A comprehensive search was conducted across three major databases: Google Scholar, PubMed, and Scopus, for publications available up to January 2024.

### 3.2. Search Strategy

A comprehensive search was performed in three major databases — Google Scholar, PubMed, and Scopus — for publications available up to January 2024. The search terms included combinations of “multiple sclerosis” with (“Natalizumab” OR “Ocrelizumab” OR “Rituximab” OR “Alemtuzumab” OR “Ofatumumab” OR “Ublituximab”) and “case report”. Relevant Medical Subject Headings (MeSH) were also incorporated. The biologic agents of interest were selected based on treatment recommendations outlined in Wolters Kluwer’s UpToDate^®^. No additional filters for date (beyond up to January 2024) or study type were applied, but limits included English language and full-text availability only.

### 3.3. Eligibility Criteria

Studies were included if they met the following criteria: (A) case reports or case series; (B) reports describing autoimmune dermatological complications associated with MS immunotherapy; and (C) availability of the full text in English. Exclusion criteria included (A) non-English publications; or (B) SADs other than dermatological disorders; (C) reviews, meta-analyses, letters to editors, clinical trials, and qualitative studies; (D) studies without patient timelines or outcomes; (E) non-biological MS therapies; and (F) incomplete case descriptions. To standardize the scope, autoimmune conditions were identified using the 2024 Autoimmune Disease List of the Global Autoimmune Institute.

### 3.4. Data Extraction

Data from the eligible studies were screened and extracted using EndNote^®^ version 7 (Clarivate Analytics). Two independent researchers reviewed the data to ensure accuracy and consistency, with the findings subsequently cross-verified by two additional team members.

### 3.5. Quality Assessment

The methodological quality of the included case reports was assessed using the Joanna Briggs Institute (JBI) critical appraisal checklist (15). This tool assesses eight Likert key aspects such as patient demographics, clinical history, diagnostic approaches, therapeutic interventions, and post-treatment outcomes of case reports. Each case report was systematically reviewed to determine its methodological robustness. The assessment also included an evaluation of the overall utility of the case reports, as detailed in Table 1. 

**Table 1.** Quality Assessment of Case Reports Using the Joanna Briggs Institute Critical Appraisal Checklist

Authors	References No.	Were Patients’ Demographic Characteristics Clearly Described?	Was the Patient’s History Clearly Described and Presented as a Timeline?	Was the Current Clinical Condition of the Patient on Presentation Clearly Described?	Were Diagnostic Tests or Assessment Methods and the Results Clearly Described?	Was the Intervention(s) or Treatment Procedure(s) Clearly Described?	Was the Post-intervention Clinical Condition Clearly Described?	Were Adverse Events (Harms) or Unanticipated Events Identified and Described?	Does the Case Report Provide Takeaway Lessons?	Overall Appraisal
**Molazadeh et al., 2021**	(16)	Y	Y	Y	Y	Y	Y	Y	Y	Include
**Tzanetakos et al., 2022**	(17)	Y	Y	Y	Y	Y	Y	Y	Y	Include
**Jakob Brecl et al., 2022**	(18)	Y	Y	Y	Y	N	Y	Y	Y	Include
**Dikeoulia et al., 2021**	(19)	Y	Y	Y	Y	UC	N	Y	Y	Include
**Darwin et al., 2018**	(20)	Y	Y	Y	Y	Y	Y	Y	N	Include
**Zimmermann et al., 2017**	(21)	Y	Y	Y	Y	UC	Y	Y	Y	Include
**Borriello et al., 2021**	(22)	Y	Y	Y	Y	Y	Y	Y	Y	Include
**Alcala et al., 2019**	(23)	Y	Y	Y	Y	N	UC	Y	Y	Include
**Naranjo Guerrero et al., 2023**	(24)	Y	Y	Y	Y	Y	Y	Y	Y	Include
**Chan et al., 2019**	(25)	Y	Y	Y	Y	Y	Y	Y	Y	Include
**Ruck et al., 2018**	(26)	Y	Y	Y	Y	N	N	Y	UC	Include
**Bolton et al., 2020**	(27)	Y	Y	Y	Y	Y	UC	Y	Y	Include
**Millan-Pascual et al., 2012**	(28)	Y	Y	UC	Y	Y	Y	Y	Y	Include
**Vacchiano et al., 2018**	(29)	Y	Y	Y	UC	N	Y	Y	Y	Include
**Lappi et al., 2022**	(30)	Y	Y	Y	Y	Y	UC	Y	Y	Include
**Tsourdi et al., 2015**	(31)	Y	Y	Y	N	Y	Y	UC	Y	Include
**Leussink et al., 2018**	(32)	Y	Y	Y	Y	Y	UC	Y	Y	Include
**Durcan et al., 2019**	(33)	Y	Y	Y	Y	Y	Y	Y	Y	Include
**Bohm et al., 2021**	(34)	Y	Y	Y	Y	UC	N	Y	Y	Include

## 4. Results

**Figure 1.**
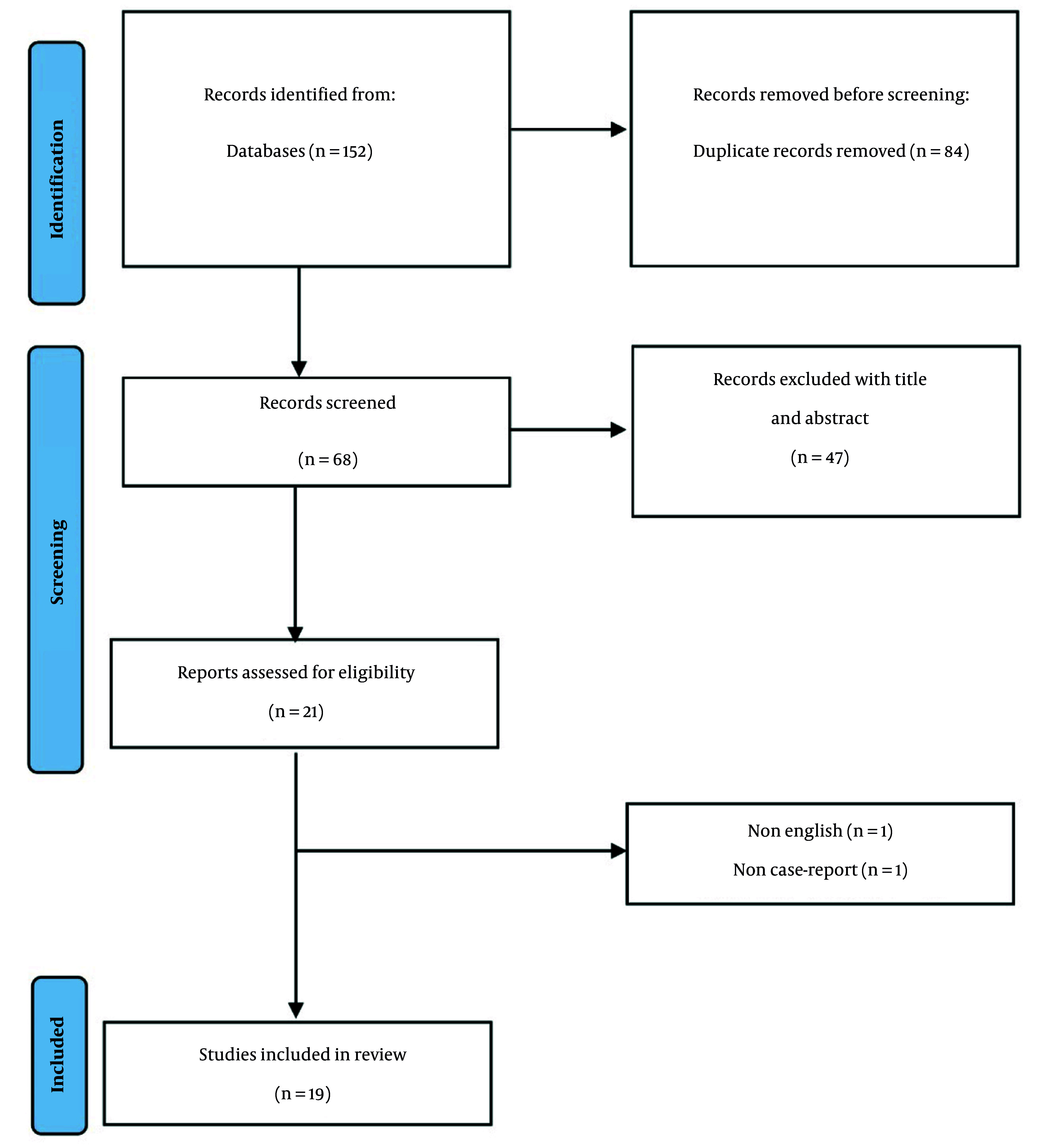
Flowchart of the included studies

A comprehensive systematic search of the specified databases through January 17, 2024, identified 152 articles. Following the removal of 84 duplicate entries, 68 articles remained for independent evaluation by two researchers. Title and abstract screening resulted in the exclusion of 47 articles. Subsequent full-text review of the remaining studies led to the elimination of two additional articles based on language restrictions and study design criteria (Figure 1). Finally, 19 articles met eligibility requirements, documenting 26 patients’ clinical details. These reports originated primarily from Germany, Spain, and Italy, with additional cases from Canada, Greece, Ireland, the UK, Iran, the USA, and Croatia. The included studies are summarized in Table 2. Ten studies examined autoimmune dermatologic complications of alemtuzumab in MS patients, while natalizumab and ocrelizumab were each the subject of 4 studies, with the remainder documenting rituximab-related effects. In total, 26 patients from 19 studies involving 4 distinct injectable biological MS therapies were identified.

**Table 2.** Case Reports of Dermatological Autoimmune Disease Induced by Multiple Sclerosis Biologic Pharmacotherapies

Drugs and Study	Type of Autoimmunity	Medical History	Timeline of Occurrence	Treatment	Outcome/MS Treatment	Demographic Characteristics	Year of Occurrence/Country	Target
**Alemtuzumab**								
Ruck et al., 2018 (26)	Vitiligo	MS since 2004	52 months after initiation, 10 months after 2nd infusion	NM	NM	A 31 year-old woman	2016/Germany	RRMS
Ruck et al., 2018 (26)	Vitiligo	MS Since 2001, fingolimod	18 months after initiation (6 month after the 2nd dose)	NM	NM	A 34 year-old A man	2017/Germany	MS
Ruck et al., 2018 (26)	Vitiligo	MS since 2015, fingolimod	14 months after initiation (2 month after the 2nd dose)	NM	NM	A 42 year-old woman	2018/Germany	MS
Bohm et al., 2021 (34)	Halo naevus-like hypopigmentation	MS since 2016	11 months after 2nd infusion	NM	NM	A 33 year-old male	2018/Germany	Highly reactive RRMS
Alcala et al., 2019 (23)	Alopecia areata	MS since 4 years ago /fingolimod	9 months after the 2nd cycle	Interlesional steroids	Improved but new plaques occured again/NM	A 28 year-old woman	2017/Spain	Aggressive RRMS
Dikeoulia et al., 2021 (19)	Alopecia areata	MS since 2006	18 months after 2nd infusion	Topical clobetasole then topical immunotherapy	NM	A 31 year-old woman	2019/ Germany	RRMS
Tsourdi et al., 2015 (31)	Alopecia areata with hyperthyroidism	MS since 2004, smoker	34 months after infusion	Topical mometasone but not effective	Thyroidectomy/NM	A 34 year old women	2012/Germany	MS
Chan et al., 2019 (25)	Alopecia areata	MS since 2015	2 months after 2nd course	Interlesional triamcinolone then IV methylprednisolone	Improved and regrowth/NM	A 31 year-old woman	2017/Canada	RRMS
Alcala et al., 2019 (23)	Alopecia universalis	MS since 2005; With history of vitiligo	5 months after 2nd cycle	Not stated	His vitiligo too was worsened/NM	A 27 year-old man	2017/Spain	Aggressive RRMS
Borriello et al., 2021 (22)	Alopecia universalis with Hashimoto’s thyroiditis	NM	12 months after 2nd dose	Topical minoxidil and retinoic acid/low vid levels	No improvement/NM	A 32 year-old woman	2019/Italy	MS
Borriello et al., 2021 (22)	Alopecia universalis with swelling in her hand	MS since 2015	12 months after 2nd dose	IV steroids+pimecrolimus+betamethasone	No hair growth/NM	A 36 year-old woman	2018/Italy	MS
Leussink et al., 2018 (32)	Alopecia universalis	MS since 2014	6 months after the last infusion	No intervention	Regrowth after 9 months/NM	A 29 year-old woman	2015/Germany	Highly active RRMS
Tzanetakos et al., 2022 (17)	Alopecia universalis with transient accommodation spasm	MS since 2005	Fingolimod/8 months after initiation	IV and oral steroid	Roughly one year after Partial hair regrowth/continued	A 24 year-old man	2018/Greece	MS
Zimmermann et al., 2017 (21)	Alopecia universalis	NM; Had received mitoxantrone before	6 months after 2nd cycles	WHO-UMC probable/likely/not consented to any therapy	No improvement /NM	A 49 year-old man	NM/Germany	RRMS
**Natalizumab**								
Durcan et al., 2019 (33)	Cutaneous sarcoidosis-like reaction	MS since 2017	Following 4th dose	Topical steroids	Poorly responsive/after 8 weeks subsided/discontinued	A 41 year-old woman	2019/Ireland	MS
Bolton et al., 2020 (27)	Cutaneous lupus erythematosus with positive anti rho ab	NM	Following 2nd infusion	Oral and topical steroid then to MMF	Rash improved/NM	A 51 year-old man	NM/UK	MS
Millan-Pascual et al., 2012 (28)	Psoriasis (reactivation)	NM; Topical agents + MTX	After 6th infusion	UVB + topical	Slight resolution/continued	A 31 year-old woman	NM/Spain	RRMS
Vacchiano et al., 2018 (29)	Arthritic psoriasis	20 years history of MS; Positive family history for psoriasis	Skin lesions, after 19th infusion and a month later, arthritis	Steroid	Partially effective/changed to DMF	A 56 year-old woman	NM/Italy	RRMS
**Ocrelizumab**								
Lappi et al., 2022 (30)	Palmoplantar pustular psoriasis	NM	3 months after the last dose	Treatment with UVB and calcipotriol/betamethasone	Complete resolution/NM	A 38 year-old woman	NM/Italy	Highly active MS
Darwin et al., 2018 (20)	Psioriasiform dermatatis	MS diagnosed at 45/trigeminal neurolgia	3.5 months after 2st infusion (end of induction)	5 on Naranjo Scale/terbinafine then clobetazole	Improvement /continued	A 68 year-old woman	2018/USA	MS
Naranjo Guerrero et al., 2023 (24)	Psioriasiform dermatatis/ fingernails was involved	NM	11 months after 2nd dose	Clobetazole/partially responsive	NM/not discontinued	A 33 year-old man	NM/Spain	RRMS
Naranjo Guerrero et al., 2023 (24)	Psioriasiform dermatatis	NM	4 months after 1st dose	Clobetazole topical/complete response	NM/not discontinued	A 36 year-old woman	NM/Spain	RRMS
Naranjo Guerrero et al., 2023 (24)	Psioriasiform dermatatis	NM	5 months after 1st dose	Calcipo/betamethasone	NM/not discontinued	A 45 year-old woman	NM/Spain	RRMS
Jakob Brecl et al., 2022 (18)	Psoriasis	MS since 2020	6 months after 1st cycle	NA oral and topical	Moderate improvement/discontinued	A 40 year-old woman	2021/Croatia	PPMS
Jakob Brecl et al., 2022 (18)	Psoriasis	MS since 2019; HTN and asthma	1 month after 2nd cycle	NA topical	Partially regressed/discontinued	A 66 year-old woman	2020/Croatia	PPMS
**Rituximab**								
Molazadeh et al., 2021 (16)	Psoriasis	MS since 2005 with migraine, bipolar disorder, seizure	2 months after 4th cycle	Topical steroids	Improved/continued	A 39 year- woman	2020/Iran	MS

Abbreviations: MS, multiple sclerosis; RRMS, relapsing-remitting multiple sclerosis; PPMS, primary progressive multiple sclerosis.

### 4.1. Alemtuzumab

Alemtuzumab represents a monoclonal antibody that targets CD52 and has demonstrated efficacy as a therapeutic option for chronic lymphocytic leukemia (CLL) and other lymphoid malignancies (35). Studies have shown its effectiveness in diminishing relapse frequency and decelerating disability progression in MS, as well as demonstrating positive effects on radiological parameters (36). However, its clinical use correlates with the emergence of acquired autoimmune conditions, particularly thyroid-associated disorders, requiring vigilant monitoring and clinical management (37).

#### 4.1.1. Autoimmune Complications

The autoimmune adverse reactions most frequently linked with alemtuzumab encompass thyroid disorders, manifesting in 20 - 30% of cases, with Graves’ disease being the predominant clinical presentation (38). Immune thrombocytopenia, hemolytic anemia, hepatitis, encephalitis, myasthenia gravis, Lambert-Eaton myasthenic syndrome, sarcoidosis, vitiligo, alopecia, myositis, and type 1 diabetes are additional autoimmune sequelae of this therapeutic agent (39). The emergence of these complications appears to correlate with the drug’s mechanism of action, which induces sustained lymphopenia and subsequent lymphocyte repertoire reconstitution (10).

### 4.2. Natalizumab

Natalizumab functions as a monoclonal antibody utilized in relapsing-remitting multiple sclerosis (RRMS) management (40). It selectively binds to the α4 subunit of α4β1 and α4β7 integrin receptors, thereby inhibiting α4-mediated leukocyte attachment to their corresponding counter-receptors (41). This mechanism prevents T-cell lymphocyte migration into the CNS, thereby reducing inflammation and neurological damage characteristic of MS.

#### 4.2.1. Autoimmune Complications

Autoimmune hepatitis (42, 43), immune thrombocytopenic purpura (44), and rheumatoid arthritis (45) have been documented with natalizumab therapy. Proposed pathophysiological mechanisms include a transition toward Th17-mediated inflammatory responses while concurrently blocking Th1 cell entry (45); however, further investigation is warranted to elucidate the underlying mechanisms and identify potential risk factors for these adverse effects.

### 4.3. Ocrelizumab

Ocrelizumab represents a humanized, second-generation, anti-CD20 monoclonal antibody that has been used in RRMS and early primary progressive multiple sclerosis (PPMS). Through binding to CD20 protein expressed on B-cells, it achieves B-cell depletion, thus preventing attacks on the CNS (46).

#### 4.3.1. Autoimmune Complications

Ocrelizumab therapy has been correlated with an elevated risk for psoriasis development and inflammatory bowel disease (IBD) (47). Furthermore, glomerulosclerosis and Graves’ disease cases have been documented (48, 49). The precise mechanism remains unclear, though some hypotheses suggest a potential association with immune system dysregulation via B-cell depletion (50).

### 4.4. Rituximab

Rituximab, a chimeric monoclonal antibody that targets CD20-expressing B-cells, has been utilized in RRMS treatment (51). In comparison to rituximab, ocrelizumab binds to a distinct but overlapping CD20 epitope (52).

#### 4.4.1. Autoimmune Complications

Several reports document rituximab-induced psoriasis (16) and ulcerative colitis (53) in MS patients. The proposed mechanism involves elimination of B-cell regulatory function that normally controls excessive T-cell activation (16).

## 5. Discussion

This systematic review of case reports constitutes the initial comprehensive evaluation of case reports examining secondary autoimmune dermatological disorders precipitated by biological immunotherapy agents utilized in MS treatment. We systematically analyzed 19 articles meeting inclusion criteria, encompassing 2 studies from the Americas, 16 from Europe, and 1 from Asia. Among these, ten studies examined alemtuzumab, which correlated with autoimmune dermatological complications in 14 patients. Four studies documented natalizumab, with 4 patients experiencing dermatological adverse reactions, while four additional studies addressed ocrelizumab-related dermal complications, affecting 7 patients. One case study described a single patient who developed secondary autoimmune dermatological disorders associated with rituximab. In total, 26 patients were identified with autoimmune dermatologic adverse effects. Notably, we found no studies documenting autoimmune dermatological complications with ofatumumab or ublituximab, two recently approved monoclonal antibodies for MS treatment, with the majority of complications attributed to alemtuzumab. Various autoimmune mechanisms have been proposed by the reviewed case reports, which are outlined in Table 3. 

**Table 3.** Different Mechanisms Proposed

Agents and Disorders	Findings and Proposed Pathogenesis
**Alemtuzumab**	
Vitiligo and halo naevus-like hypopigmentation	Melanocytes destruction of not-depleted melanocyte-specific CD8+ T-cells (34); Rise in interleukin-21 which drives proliferation of chronically activated, oligoclonal, effector memory T-cells (26); Increment in anti-tyrosinase antibodies and sharp rise in antibodies against tyrosinase -related protein 1 (34)
Alopecia areata and Alopecia universalis	Profound immunosuppression followed by immune cell reconstitution leads to an increased number of T and B lymphocytes and anti-inflammatory cytokines leading to auto reactivity and reduced self-tolerance (23); The unregulated expansion of the B-cell pool, and increase levels of B-cell activating factor leading to uncontrolled autoantibody production (22); Escaped peripheral T-cells proliferate to restore the T repertoire plus over-expression of cytokines, reduced thymic output, and the Treg/non-Treg ratio skewing (22); Immune reconstitution after immunosuppression of alemtuzumab, during which B-cells recover more rapidly than T-cells, resulting in insufficient T-cell regulation, leading to uncontrolled B-cell autoreactivity (19); Lower vitamin D levels leading to rise in interleukin-21 and -17 inducing Th17 and inhibit re-differentiation of regulatory T-cell (22)
**Natalizumab**	
Cutaneous sarcoidosis-like reaction	Altering expression of the α4β1− integrin, surrounding lymphocytes and macrophages of sarcoid granulomas which changes the structural extracellular matrix, inducing an inflammatory cascade and subsequent formation of granulomata (33)
Cutaneous lupus erythematosus	Apoptosis elevated rates are with α4β1-integrin interactions’ interference, leading to presentation of autoantigens and formation of autoantibodies (27); Disrupted signaling of α4β1-integrin important to T-cell progenitors’ thymic selection (27); Suppressed α4β1-integrin activation related to decrease in the differentiation and suppressive function of peripheral T regulatory cells (27)
Psoriasis	In chronic inflammation, proinflammatory cytokines’ dysregulated production leads to adhesion molecules expression rise, blockade of one of these molecules can be compensated for by other pathways (28)
Arthritic psoriasis	Altering laminin function (29)
**Ocrelizumab**	
Psioriasiform dermatatis	Depletion of regulatory B‐lymphocytes with immunomodulatory function through interleukin‐10 and proinflammatory cytokines release such as tumor necrosis factor‐α, interleukin-6 and -8 (24)
Psoriasis	B-cell depletion stops the B-cells regulatory effect on T-cells population (30); Preceding the release of TNF-alpha, IL-6 and IL-8, promoting angiogenesis, generating a pro-inflammatory environment, inducing keratinocyte proliferation, and attracting neutrophils (30); Increment the susceptibility to bacterial infection and modification of the microbiome (30)
**Rituximab**	
Psoriasis	Removal of B-cell regulatory function by controlling excessive T-cell activity (16); Induce complement activation (16)

Several patients have been documented with psoriasis development during interferon beta (IFNB) therapy, with some cases showing plaque formation at injection sites. This has been theorized to involve stimulation of the interleukin-23-Th17 pathway, associated with psoriasis pathogenesis, activating granulocyte recruitment and proinflammatory factor release in dermal tissue (28). Interleukin-17 is recognized for inhibiting keratinocyte proliferation and differentiation while simultaneously promoting Th17 cell recruitment, which produces additional interleukin-17, creating a positive feedback loop associated with psoriatic inflammatory responses (29). Documentation of new arthritis during IFNB treatment was also published in 2010 (29). One patient developed alopecia following alemtuzumab during the 4 - 5 year follow-up of 61 patients with high disease activity MS (21). Two individuals in another observational study of 100 patients followed for 6.2 years experienced alopecia characterized as an autoimmune complication after alemtuzumab treatment (54). The pathophysiological basis of alemtuzumab-induced autoimmunity remains inadequately understood. Given the high prevalence of antibody-mediated autoimmune complications, B-cells are presumed to be primary mediators (26). Additionally, chronic activation and proliferation of oligoclonal, effector memory CD8+ T-cells represents one hypothesis explaining this phenomenon (34). Another study proposed that following initial lymphopenia induction (1) The subsequent expansion of T-cells reactive to self-antigens that escaped depletion, combined with increased likelihood of self-antigen encounter and/or; (2) The subsequent B-cell increase are responsible for autoimmune disease secondary to alemtuzumab use (25). Furthermore, analysis demonstrated an increased secondary autoimmunity risk in patients treated with alemtuzumab who previously received fingolimod (17).

Natalizumab specifically targets the α4 integrin subunit, present on both α4β7 and α4β1, also known as very-late antigen-4 (VLA-4). While leukocyte endothelial adherence represents the primary integrin function, α4-integrins also contribute to tissue-specific lymphocyte trafficking in both physiological and pathological contexts. Their blockade has demonstrated paradoxical exacerbation in animal IBD models. The VLA-4 may be crucial for lymphocyte CNS trafficking. In inflammatory conditions like MS, the VLA-4 ligand, VCAM-1, is substantially increased in CNS microvessels (28). Similar to natalizumab, vedolizumab, a monoclonal antibody targeting α4β7 integrin, was associated with systemic lupus erythematosus (SLE) during treatment (27). Efalizumab, targeting the integrin αL subunit, has been implicated in autoimmune disorder development such as lupus-like syndrome (28).

Psoriasiform dermatitis induced by anti-CD20 therapy has been documented in literature, primarily associated with the chimeric monoclonal antibody, rituximab (24). A descriptive study conducted in the United States indicated that psoriasiform dermatitis incidence linked to B-lymphocyte-depleting MS treatments was significantly higher than that associated with other pharmaceutical therapies for this condition (55). Complement activation is less pronounced with ocrelizumab compared to rituximab, which may explain why psoriasis has been documented so infrequently with this medication (30). Understanding the specific mechanisms by which these drugs may trigger SADs is crucial for devising strategies to mitigate these complications and optimize patient care.

### 5.1. Conclusions

Multiple sclerosis represents one of the most challenging conditions to manage clinically. While novel interventions continue to emerge for symptom control, safety profiles — including dermatological SADs — remain critical, as evidenced by comparisons with other MS-related research on immune modifications and comorbidities. As additional novel medical interventions are commercialized to control its symptoms and complications, more safety reports are published. Secondary dermatological autoimmune disorders constitute some of the many documented adverse effects. A comprehensive review article addressing these novel side effects was lacking. We attempted to review reports on this issue to explain new aspects and help physicians better understand the problems their patients might encounter.

### 5.2. Limitations

Due to their high risk of bias, case reports and case series are often considered weak evidence sources. Additionally, language restrictions prevented us from reviewing some articles.

## Data Availability

The dataset presented in the study is available on request from the corresponding author during submission or after publication.

## References

[1] Rodriguez Murua S, Farez MF, Quintana FJ (2022). The Immune Response in Multiple Sclerosis.. Annu Rev Pathol..

[2] Wootla B, Eriguchi M, Rodriguez M (2012). Is multiple sclerosis an autoimmune disease?. Autoimmune Dis..

[3] Eftekharian MM, Mousavi M, Hormoz MB, Roshanaei G, Mazdeh M (2014). Multiple sclerosis and immunological-related risk factors: results from a case-control study.. Hum Antibodies..

[4] Trapp BD, Nave KA (2008). Multiple sclerosis: an immune or neurodegenerative disorder?. Annu Rev Neurosci..

[5] Chouhfeh L, Kavak KS, Teter BE, Weinstock-Guttman B (2015). Disease modifying therapies use associated with comorbid autoimmune diseases in multiple sclerosis patients.. Mult Scler Relat Disord..

[6] Ghiasian M, Lashkari N, Mohammadi M, Mohammadi Y, Soleimani M, Mahjub R (2024). Assessment of Total Side Effects of Oral Agents for the Treatment of Relapsing–Remitting Multiple Sclerosis Patients in Imam Khomeini Clinic in Hamadan.. Armaghane Danesh..

[7] Sahraian MA, Salehi AM, Jenabi E, Esfahani ME, Ataei S (2022). Post marketing new adverse effects of oral therapies in multiple sclerosis: A systematic review.. Mult Scler Relat Disord..

[8] Naderi N (2015). The Perspectives of Mesenchymal Stem Cell Therapy in the Treatment of Multiple Sclerosis.. Iran J Pharm Res..

[9] Soleimani B, Murray K, Hunt D (2019). Established and Emerging Immunological Complications of Biological Therapeutics in Multiple Sclerosis.. Drug Saf..

[10] Costelloe L, Jones J, Coles A (2012). Secondary autoimmune diseases following alemtuzumab therapy for multiple sclerosis.. Expert Rev Neurother..

[11] Wildner P, Selmaj KW (2017). Multiple sclerosis: Skin-induced antigen-specific immune tolerance.. J Neuroimmunol..

[12] Mansourzadeh A, Shaygannejad V, Mirmosayyeb O, Afshari-Safavi A, Gay MC (2023). Effectiveness of Schema Therapy on Anxiety, Depression, Fatigue, Quality of Life, and Sleep in Patients with Multiple Sclerosis: A Randomized Controlled Trial.. Middle East J Rehabil Health Stud..

[13] Emami Razavi SZ, Azadvari M, Mirmosayyeb O, Vaheb S, Ghajarzadeh M, Teymouri A (2025). Botulinum Toxin Injection for Treating Spasticity in Multiple Sclerosis Patients: A Systematic Review and Meta-Analysis.. Middle East J Rehabil Health Stud..

[14] Rahmatian A, Rizehbandi M, Bastani E, Modara F, Shokri F (2024). Investigating the State of Sleep Disorders and the Factors Affecting Them in Patients with Multiple Sclerosis: Cross-Sectional Study.. Arch Neurosci..

[15] Munn Z, Barker TH, Moola S, Tufanaru C, Stern C, McArthur A (2020). Methodological quality of case series studies: an introduction to the JBI critical appraisal tool.. JBI Evid Synth..

[16] Molazadeh N, Ala S, Karaminia M, Sahraian MA (2021). Rituximab induced psoriasis in a patient with multiple sclerosis: A case report and literature review.. Neuroimmunol Rep..

[17] Tzanetakos D, Breza M, Tzartos JS, Bontzos G, Vakrakou AG, Dermentzoglou A (2022). Alemtuzumab-induced alopecia universalis and transient accommodation spasm in a patient with multiple sclerosis.. Ther Adv Neurol Disord..

[18] Jakob Brecl G, Gabelic T, Ruska B, Horvat Ledinek A, Habek M (2022). Psoriasis caused by ocrelizumab in two persons with primary progressive multiple sclerosis.. Int J Dermatol..

[19] Dikeoulia E, Neufeld M, Pawlitzki M, Böhm M (2021). Alemtuzumab‐induced Alopecia areata – a case report and systematic literature review of adverse events associated with Alemtuzumab.. JDDG..

[20] Darwin E, Romanelli P, Lev-Tov H (2018). Ocrelizumab-induced psoriasiform dermatitis in a patient with multiple sclerosis.. Dermatol Online J..

[21] Zimmermann J, Buhl T, Muller M (2017). Alopecia Universalis following Alemtuzumab Treatment in Multiple Sclerosis: A Barely Recognized Manifestation of Secondary Autoimmunity-Report of a Case and Review of the Literature.. Front Neurol..

[22] Borriello G, Ianniello A, Toosy AT (2021). Alopecia Universalis Occurring after Alemtuzumab Treatment for Multiple Sclerosis. A Two-Year Follow-Up of Two Patients.. Int J Environ Res Public Health..

[23] Alcala C, Pzere-Miralles F, Gascon F, Evole M, Estutia M, Gil-Perotin S (2019). Recurrent and universal alopecia areata following alemtuzumab treatment in multiple sclerosis: A secondary autoimmune disease.. Mult Scler Relat Disord..

[24] Naranjo Guerrero N, González Quesada A, García Minarro A, Castro González E, Paredes Pérez AB, Carretero Hernández G (2023). Ocrelizumab‐induced psoriasiform dermatitis: Case reports and review of the literature.. JEADV Clinical Practice..

[25] Chan JK, Traboulsee AL, Sayao AL (2019). Case of alemtuzumab-related alopecia areata management in MS.. Neurol Neuroimmunol Neuroinflamm..

[26] Ruck T, Pfeuffer S, Schulte-Mecklenbeck A, Gross CC, Lindner M, Metze D (2018). Vitiligo after alemtuzumab treatment: Secondary autoimmunity is not all about B-cells.. Neurol..

[27] Bolton C, Mouyis K, Bhamra K, Steuer A (2020). The first case of natalizumab-induced subacute cutaenous lupus erythematosus.. Rheumatol..

[28] Millan-Pascual J, Turpin-Fenoll L, Del Saz-Saucedo P, Rueda-Medina I, Navarro-Munoz S (2012). Psoriasis during natalizumab treatment for multiple sclerosis.. J Neurol..

[29] Vacchiano V, Foschi M, Sabattini L, Scandellari C, Lugaresi A (2018). Arthritic psoriasis during natalizumab treatment: a case report and review of the literature.. Neurol Sci..

[30] Lappi A, Cammarata E, Nicola S, Borrelli P (2022). Palmoplantar pustular psoriasis induced by ocrelizumab in a patient affected by multiple sclerosis.. Ital J Dermatol Venerol..

[31] Tsourdi E, Gruber M, Rauner M, Blankenburg J, Ziemssen T, Hofbauer LC (2015). Graves' disease after treatment with alemtuzumab for multiple sclerosis.. Hormones..

[32] Leussink VI, Reifenberger J, Hartung H (2018). Case of alopecia universalis associated with alemtuzumab treatment in MS.. Neurol Neuroimmunol Neuroinflamm..

[33] Durcan R, Heffron C, Sweeney B (2019). Natalizumab induced cutaneous sarcoidosis-like reaction.. J Neuroimmunol..

[34] Bohm M, Kemp EH, Metze D, Muresan AM, Neufeld M, Luiten RM (2021). Alemtuzumab-induced halo naevus-like hypopigmentation - new insights into secondary skin autoimmunity in response to an immune cell-depleting antibody.. J Eur Acad Dermatol Venereol..

[35] Katsavos S, Coles A (2018). Alemtuzumab as Treatment for Multiple Sclerosis.. Cold Spring Harb Perspect Med..

[36] Willis MD, Robertson NP (2016). Alemtuzumab for Multiple Sclerosis.. Curr Neurol Neurosci Rep..

[37] Kousin-Ezewu O, Coles A (2013). Alemtuzumab in multiple sclerosis: latest evidence and clinical prospects.. Ther Adv Chronic Dis..

[38] Guarnera C, Bramanti P, Mazzon E (2017). Alemtuzumab: a review of efficacy and risks in the treatment of relapsing remitting multiple sclerosis.. Ther Clin Risk Manag..

[39] Killestein J, van Oosten B (2019). Emerging safety issues in alemtuzumab-treated MS patients.. Mult Scler..

[40] Hutchinson M (2007). Natalizumab: A new treatment for relapsing remitting multiple sclerosis.. Ther Clin Risk Manag..

[41] Khoy K, Mariotte D, Defer G, Petit G, Toutirais O, Le Mauff B (2020). Natalizumab in Multiple Sclerosis Treatment: From Biological Effects to Immune Monitoring.. Front Immunol..

[42] Martinez-Lapiscina EH, Lacruz F, Bolado-Concejo F, Rodriguez-Perez I, Ayuso T, Garaigorta M (2013). Natalizumab-induced autoimmune hepatitis in a patient with multiple sclerosis.. Mult Scler..

[43] Lisotti A, Azzaroli F, Brillanti S, Mazzella G (2012). Severe acute autoimmune hepatitis after natalizumab treatment.. Dig Liver Dis..

[44] Stosic M, De Jesus P, McCarthy J, Hutton G, Rivera V (2011). Immune thrombocytopenic purpura in a patient with multiple sclerosis treated with natalizumab.. Neurol..

[45] Su E, Novic J, Han MH (2020). Emergence of rheumatoid arthritis following exposure to natalizumab.. Mult Scler Relat Disord..

[46] Lamb YN (2022). Ocrelizumab: A Review in Multiple Sclerosis.. Drugs..

[47] Mancinelli CR, Rossi N, Capra R (2021). Ocrelizumab for the Treatment of Multiple Sclerosis: Safety, Efficacy, and Pharmacology.. Ther Clin Risk Manag..

[48] Duarte DB, Silva AMD, Freitas C, Cardoso H (2021). Graves' disease with spontaneous resolution following ocrelizumab in primary progressive multiple sclerosis.. Endocr Regul..

[49] Greve AS, Prakash S, Krag S, Randers E (2023). Focal segmental glomerulosclerosis in a patient with multiple sclerosis treated with Teriflunomide and Ocrelizumab.. J Nephrol..

[50] Lee HH, Sritharan N, Bermingham D, Strey G (2020). Ocrelizumab-Induced Severe Colitis.. Case Rep Gastrointest Med..

[51] Chisari CG, Sgarlata E, Arena S, Toscano S, Luca M, Patti F (2022). Rituximab for the treatment of multiple sclerosis: a review.. J Neurol..

[52] Sorensen PS, Blinkenberg M (2016). The potential role for ocrelizumab in the treatment of multiple sclerosis: current evidence and future prospects.. Ther Adv Neurol Disord..

[53] Shahmohammadi S, Sahraian MA, Shahmohammadi A, Doosti R, Zare-Mirzaie A, Naser Moghadasi A (2018). A presentation of ulcerative colitis after rituximab therapy in a patient with multiple sclerosis and literature review.. Mult Scler Relat Disord..

[54] Willis MD, Harding KE, Pickersgill TP, Wardle M, Pearson OR, Scolding NJ (2016). Alemtuzumab for multiple sclerosis: Long term follow-up in a multi-centre cohort.. Mult Scler..

[55] Porwal MH, Patel D, Maynard M, Obeidat AZ (2022). Disproportional increase in psoriasis reports in association with B cell depleting therapies in patients with multiple sclerosis.. Mult Scler Relat Disord..

