# Erratum: Secondary Autoimmune Dermatological Disorders Induced by Multiple Sclerosis Biological Immunotherapy Agents: A Systematic Review of Case Reports [IJ Pharm Res. 2025; 24 (1): e166426]

**DOI:** 10.5812/ijpr-169319

**Published:** 2025-12-28

**Authors:** Mohammad Ali Sahraian, Shahaboddin Emami, Sara Ataei, Fahime Nasr Esfahani, Nasibeh Ghalandari

**Affiliations:** 1 Department of Neurology, Multiple Sclerosis Research Center, Neuroscience Institute, Sina Hospital, School of Medicine, Tehran University of Medical Sciences, Tehran, Iran; 2 Department of Clinical Pharmacy, School of Pharmacy, Hamadan University of Medical Sciences, Hamadan, Iran; 3 Medicinal Plants and Natural Products Research Center, Institute of Cancer, Hamadan University of Medical Sciences, Hamadan, Iran; 4 Department of Clinical Pharmacy, School of Pharmacy, Hamadan University of Medical Sciences, Hamadan, Iran; 5 Tehran University of Medical Sciences, Tehran, Iran

Dear Readers,

Following the publication of a recently published article, an error was identified in the authorship information (1).

 The name of the second author was published incorrectly. The correct name is Shahaboddin Emami.

In addition, the second author has two affiliations. The second affiliation is as follows:

Medicinal Plants and Natural Products Research Center, Institute of Cancer, Hamadan University of Medical Sciences, Hamadan, Iran

These corrections do not affect the scientific content of the article. The authors apologize for this error and any inconvenience it may have caused to the readers.

Sincerely Yours

Nasibeh Ghalandari

Department of Clinical Pharmacy, School of Pharmacy, Hamadan University of Medical Sciences, Hamadan, Iran

The Corresponding Author

## References

[1] Sahraian MA, Emami S, Ataei S, Nasr Esfahani F, Ghalandari N (2025). Secondary Autoimmune Dermatological Disorders Induced by Multiple Sclerosis Biological Immunotherapy Agents: A Systematic Review of Case Reports.. IJ Pharm Res..

